# The polar night shift: seasonal dynamics and drivers of Arctic Ocean microbiomes revealed by autonomous sampling

**DOI:** 10.1038/s43705-021-00074-4

**Published:** 2021-12-11

**Authors:** Matthias Wietz, Christina Bienhold, Katja Metfies, Sinhué Torres-Valdés, Wilken-Jon von Appen, Ian Salter, Antje Boetius

**Affiliations:** 1 Deep-Sea Ecology and Technology, Alfred Wegener Institute Helmholtz Centre for Polar and Marine Research, Bremerhaven, Germany; 2 Max Planck Institute for Marine Microbiology, Bremen, Germany; 3 Polar Biological Oceanography, Alfred Wegener Institute Helmholtz Centre for Polar and Marine Research, Bremerhaven, Germany; 4 Marine BioGeoScience, Alfred Wegener Institute Helmholtz Centre for Polar and Marine Research, Bremerhaven, Germany; 5 Physical Oceanography of the Polar Seas, Alfred Wegener Institute Helmholtz Centre for Polar and Marine Research, Bremerhaven, Germany; 6 Faroe Marine Research Institute, Tórshavn, Faroe Islands; 7 MARUM Center for Marine Environmental Sciences, University of Bremen, Bremen, Germany

**Keywords:** Microbial ecology, Microbial biooceanography, Biogeochemistry

## Abstract

The Arctic Ocean features extreme seasonal differences in daylight, temperature, ice cover, and mixed layer depth. However, the diversity and ecology of microbes across these contrasting environmental conditions remain enigmatic. Here, using autonomous samplers and sensors deployed at two mooring sites, we portray an annual cycle of microbial diversity, nutrient concentrations and physical oceanography in the major hydrographic regimes of the Fram Strait. The ice-free West Spitsbergen Current displayed a marked separation into a productive summer (dominated by diatoms and carbohydrate-degrading bacteria) and regenerative winter state (dominated by heterotrophic Syndiniales, radiolarians, chemoautotrophic bacteria, and archaea). The autumn post-bloom with maximal nutrient depletion featured *Coscinodiscophyceae*, *Rhodobacteraceae* (e.g. *Amylibacter*) and the SAR116 clade. Winter replenishment of nitrate, silicate and phosphate, linked to vertical mixing and a unique microbiome that included *Magnetospiraceae* and Dadabacteriales, fueled the following phytoplankton bloom. The spring-summer succession of *Phaeocystis*, *Grammonema* and *Thalassiosira* coincided with ephemeral peaks of *Aurantivirga*, *Formosa, Polaribacter* and NS lineages, indicating metabolic relationships. In the East Greenland Current, deeper sampling depth, ice cover and polar water masses concurred with weaker seasonality and a stronger heterotrophic signature. The ice-related winter microbiome comprised *Bacillaria*, *Naviculales*, *Polarella*, *Chrysophyceae* and *Flavobacterium* ASVs. Low ice cover and advection of Atlantic Water coincided with diminished abundances of chemoautotrophic bacteria while others such as *Phaeocystis* increased, suggesting that Atlantification alters microbiome structure and eventually the biological carbon pump. These insights promote the understanding of microbial seasonality and polar night ecology in the Arctic Ocean, a region severely affected by climate change.

## Introduction

Microbes are fundamental for the marine biosphere and have been recognized as key components of global change biology [1]. Understanding the causes, complexity, and consequences of microbial community dynamics significantly benefits from continuous observations in the physicochemical context. Ocean time series are beginning to discern the temporal variability and environmental drivers of marine microbiomes from diurnal to decadal scales, but focusing on temperate and tropical waters to date [2–6]. In contrast, continuous records from the polar oceans are rare. Pioneering studies have identified variable numbers, activities, and communities of polar microbes over time and space [7–13] indicating considerable seasonal contrasts [14], yet with limited temporal or spatial resolution.

Due to the extreme winter conditions and remoteness, continuous observations covering the polar night have been seldom accomplished through shipboard expeditions, or performed in coastal areas [15, 16]. New autonomous technologies are a key advance for year-round studies in polar waters, recently providing the first annual records in the Arctic and Antarctic Oceans [17, 18]. Such approaches can identify transition phases in the seasonal interplay between ocean physics and the ecosystem, for instance the onset of the spring bloom or the end of net growth. In this regard, the polar night is of key interest, when physical mixing [19, 20] and microbial activities [21, 22] replenish nutrients to fuel the subsequent phytoplankton bloom. Arctic phototrophic taxa are thought to overwinter in dormancy [23], responding rapidly when light returns [15, 24], but recent evidence suggests that primary production might already start from late winter [18]. However, microbial dynamics in the open Arctic Ocean during the polar night, especially in presence of sea ice, remain largely unknown.

**Fig. 1:**
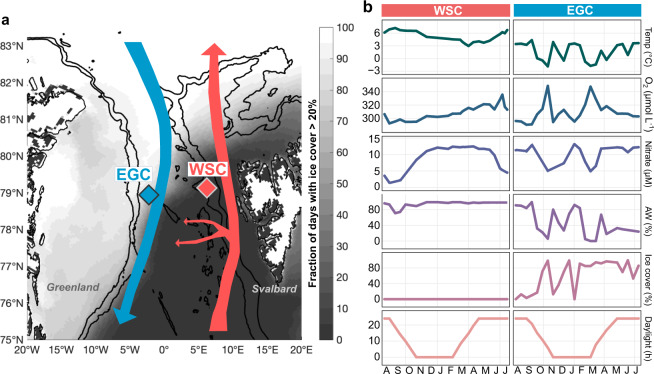
Study area and oceanographic conditions. **a** Location of moored Remote Access Samplers in the East Greenland Current (EGC) and the West Spitsbergen Current (WSC) of Fram Strait, indicated in blue and red respectively. The small red arrows illustrate recirculation of Atlantic Water in central Fram Strait. The grayscale gradient indicates the fraction of days with average sea ice cover of >20%. **b** Water temperature (°C), concentrations of oxygen (µmol L^−1^) and nitrate (µM), the proportion of Atlantic Water (%), sea ice cover (%), and daylight hours.

Here, using an array of autonomous samplers and sensors, we portray microbial and oceanographic seasonality in the two major hydrographic regimes of the Fram Strait. This main deep-water gateway to the central Arctic Ocean harbors the northward, relatively warm and ice-free West Spitsbergen Current (WSC) and the southward, ice-covered and cold East Greenland Current (EGC), with some recirculation in central Fram Strait across the marginal ice zone (Fig. 1a). Our study is embedded in the long-term HAUSGARTEN observatory studying primary production, benthopelagic coupling, and deep-sea ecology since the 1990s [25, 26]. The recent deployment of autonomous devices within the FRAM infrastructure program affords the unique opportunity for continuous year-round records. These considerably expand summertime observations of microbial diversity and activity in the WSC and EGC [27–32], shaped by a combination of sea ice cover, nitrate availability, and mixed layer depth [33, 34]. Annual records also help to understand the biological responses to the northward expansion of subarctic habitats, termed Atlantification, which propagates through the entire food web [35].

Here we investigated how polar day and night shape seasonality, expecting considerable differences between summer and winter microbiomes in both regions. We hypothesized that phototrophy- and heterotrophy-dominated periods in the WSC harbor markedly dissimilar microbial communities, whereas sea ice cover and polar water masses in the EGC sustain winter-type communities year-round. Our study illuminates fundamental principles of seasonality in Arctic microbial diversity, the ecological importance of the polar night, and potential effects of Atlantification. This evidence helps understanding natural variability and human impact in a region under severe threat by climate change [36, 37], with important implications for the present and future Arctic Ocean.

## Materials and methods

### Sampling approach

Within the framework of the FRAM marine observatory (https://www.awi.de/en/expedition/observatories/ocean-fram.html), Remote Access Samplers (RAS; McLane, East Falmouth, MA) were deployed in July 2016 on seafloor moorings F4-S-1 in the core WSC (79.0118 N 6.9648 E) and EGC-3 in the marginal ice zone (78.831 N−2.7938 E), constituting a fixed-point Eulerian approach (Fig. 1a). RAS deployment depth was 30 m (WSC) and 80 m (EGC; to avoid ice collisions). However, vertical movements in the water column resulted in variable actual sampling depths, with a mean of 40 m and 90 m in the WSC and EGC respectively (Supplementary Table 1). RAS frames were equipped with 48 sterile sampling bags, each containing 700 µL of saturated (7.5% w/v) mercuric chloride solution. At each programmed sampling event, two water samples of 500 mL were autonomously pumped an hour apart into individual sampling bags and fixed by mixing with mercuric chloride (0.01% final concentration). Upon recovery in August 2017, samples were immediately filtered through 0.22 µm Sterivex cartridges (Millipore, Burlington, MA) and frozen at –20 °C until DNA extraction.

### DNA extraction and amplicon sequencing

DNA was extracted using the PowerWater kit (QIAGEN, Germany) according to the manufacturer’s instructions, and quantified using Quantus (Promega, Madison, WI). 16S and 18S rRNA gene fragments were amplified using primers 515F–926R [38] and 528iF–964iR [29] respectively. Libraries were prepared according to the 16S Metagenomic Sequencing Library Preparation protocol (Illumina, San Diego, CA). rRNA gene fragments were sequenced using MiSeq technology in 2x300bp paired-end runs (Supplementary Methods).

### Sequence analysis

After primer removal using cutadapt [39], 16S and 18S rRNA reads were processed into amplicon sequence variants (ASVs) using DADA2 v1.14.1 [40] and classified using taxonomy databases Silva v138 [41] and PR^2^ v4.12 [42] respectively (Supplementary Methods). After singleton removal, we obtained on average 62,000 16S rRNA and 99,000 18S rRNA reads per sample (Supplementary Table 2) sufficiently covering community composition (Supplementary Fig. 1). Sequences have been deposited in the European Nucleotide Archive under accession numbers PRJEB43890 (16S rRNA) and PRJEB43504 (18S rRNA) using the data brokerage service of the German Federation for Biological Data (GFBio) in compliance with MIxS standards.

### Mooring and satellite data

Temperature, depth, salinity, oxygen concentration, and oxygen saturation were derived from Seabird SBE37-ODO CTD sensors attached to the RAS, confirming consistent properties of the two water samples per date. Sensor measurements were averaged over 4 h around each sampling event, allowing to determine the relative proportions of Atlantic Water (AW) and Polar Water (PW) (Supplementary Methods). Relative proportions of >80% were considered as pure Atlantic or Polar Water respectively; and 20–80% as mixture of both. Physical sensors were manufacturer-calibrated and processed in accordance with https://epic.awi.de/id/eprint/43137. For chemical sensors (Sunburst SAMI-pH and Sunburst SAMI-CO_2_), the raw readouts are reported. Mooring data are available under https://doi.pangaea.de/10.1594/PANGAEA.904565. Sea ice and surface chlorophyll concentrations, derived from the AMSR-2 and Sentinel 3A OLCI satellites, were downloaded from the University of Bremen and the European Space Agency respectively, considering grid points within a radius of 15 km around the moorings.

### Nutrient quantification

Nitrate, nitrite, phosphate, and silicate were quantified using a QuAAtro Seal Analytical segmented continuous-flow autoanalyser following standard colorimetric techniques. Accuracy was evaluated using KANSO LTD Japan Certified Reference Materials, with corrections applied as required. Following quality controls, results deemed questionable or of bad quality (quality flags 4 or 8 respectively) were excluded from further analyses (labeled NA in Supplementary Table 1). Nutrient data are available under https://doi.pangaea.de/10.1594/PANGAEA.936749.

### Statistical evaluation

**Fig. 2:**
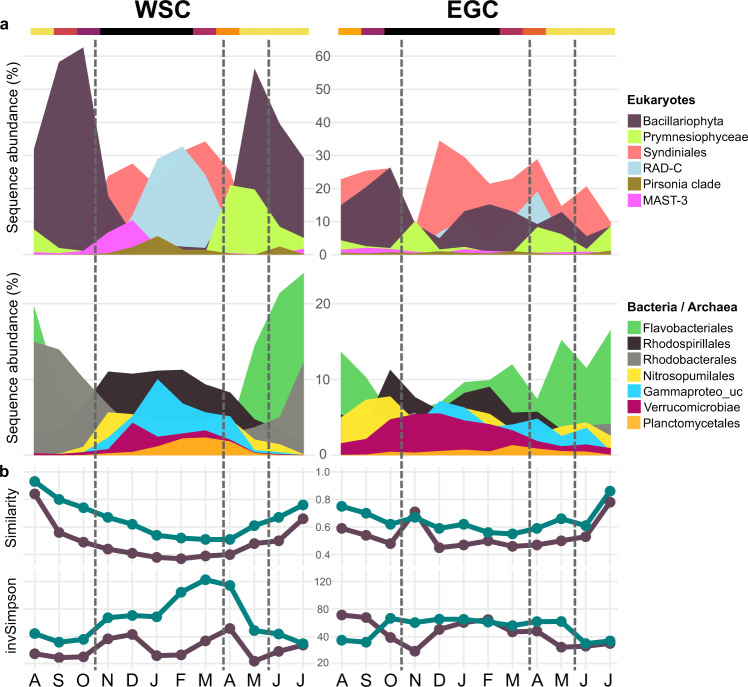
Year-round microbial community structure and turnover. **a** Relative sequence abundances (%) of eukaryotic, bacterial and archaeal taxa over the annual cycle. **b**, upper panel**:** Microbial community turnover (taxonomic similarities expressed as 1 minus Jenson-Shannon distance) compared to the first sampling event in relation to daylight hours (top color gradient). **b**, lower panel: Microbial alpha-diversity (inverse Simpson index). Eukaryotes: purple; bacteria and archaea: green. Lines indicate the seasonal boundaries defined by multivariate evaluation of physicochemical and microbial dynamics (Fig. 3).

**Fig. 3:**
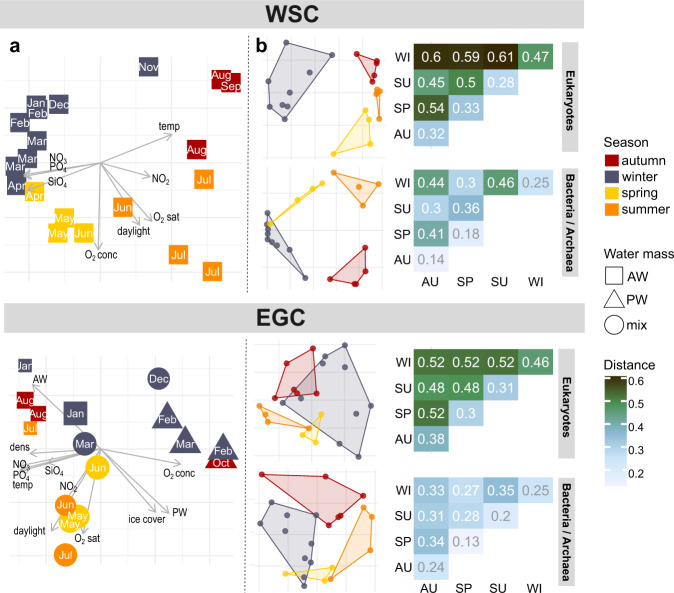
Microbial and environmental seasonality. **a** Principal Component Analysis of environmental conditions. Components 1 and 2 explained 58/26% (WSC) and 60/14% (EGC) respectively, and hence the majority of physicochemical variability. For EGC, label size indicates percent ice cover. Only sampling events with complete environmental data were considered. **b** Non-metric multidimensional scaling of Hellinger-transformed relative ASV abundances (stress values 0.07, 0.03, 0.13, 0.1 from top to bottom) and corresponding Jensen-Shannon distances between and within seasons (larger numbers designate more dissimilar communities).

Data analysis was done in R v4.1.1 implemented in RStudio (https://rstudio.com). In short, alpha-diversity and rarefaction curves were computed on raw ASV counts using R package iNEXT [43], excluding metazoan, chloroplast, and mitochondrial sequences. Subsequently, we only considered reads with ≥3 counts in ≥2 samples. Also, two samples from >200 m depth, when the RAS was pushed down by currents, were discarded to omit deep-water signatures. NMDS was performed using Bray-Curtis dissimilarities on Hellinger-transformed relative abundances. Seasons were defined based on multivariate patterning of oceanographic parameters and microbial community composition (Figs. 2, 3). Statistical differences were computed by PERMANOVA or Kruskal-Wallis plus Bonferroni-corrected Dunn’s post-hoc test as appropriate. Pairwise associations were assessed by Spearman correlations. Major R packages used were tidyverse, phyloseq, ampvis2, and PNWColors [44–47]. Code for reproducing workflow and figures is available at https://github.com/matthiaswietz/RAS-1617.

## Results and discussion

The present study elucidates microbial and oceanographic seasonality in the WSC and the EGC of Fram Strait using automated, year-round high-frequency sampling (Fig. 1a). For this purpose, seawater was autonomously collected and preserved in situ using moored Remote Access Samplers (RAS) in weekly to monthly intervals (Supplementary Table 1). In addition, sensors continuously measured depth, temperature, salinity, and oxygen, informing about oceanographic conditions including the proportions of Atlantic Water (AW) and Polar Water (PW). After recovery, water samples were subjected to amplicon sequencing of microbial communities and quantification of inorganic nutrients. Bacterial, archaeal, and eukaryotic amplicon sequence variants (ASVs) were then evaluated in the oceanographic context, including satellite-derived ice and chlorophyll concentrations (Supplementary Table 1).

### Major annual dynamics and drivers

Environmental conditions and microbial communities substantially differed over the year, but also between the two sampling sites (Fig. 1b, Supplementary Figs. 2, 3). At the WSC mooring, ice-free AW prevailed throughout the year, with water temperatures between 3.0 and 7.2 °C at sampling depth (Supplementary Table 1). Stratification in summer and mixing of the water column in winter [48, 49] corresponded to a mixed layer depth between 0 (July/August) and 270 m (February). At the EGC mooring, deployed at the edge of the marginal ice zone, water temperature varied between –1.8 and 4.4 °C. Intermittent advection of AW resulted in dynamic changes between polar (cold/ice-rich) and Atlantic (warmer/low-ice) conditions (Fig. 1b). PW-dominated periods showed a specific physicochemical and microbial signature, whereas AW advection resulted in greater similarities to the WSC (Supplementary Figs. 2, 3). This connection was strongest between AW proportions and bacterial composition (Spearman’s *rho* = 0.4; *p* = 0.00008). Hence, differences between the WSC and EGC correspond to different hydrography, ice cover as well as sampling depth. Earlier studies investigated the background of these vertical and horizontal contrasts in hydrography [33] and microbial composition [29].

This study focuses on the seasonal shifts in microbial communities. In both the WSC and EGC, communities markedly changed in composition and diversity over the annual cycle (Fig. 2a), illustrating dynamic microbiome structures year-round. Taxonomic dissimilarities to the first sampling event peaked around the March equinox before increasing again towards peak polar day (Fig. 2b), indicating light-driven temporal recurrence [50]. Notably, bacterial but not eukaryotic alpha-diversity correlated with daylight hours in both regions (Spearman’s *rho* = 0.6, *p* < 0.006).

### Microbial and environmental seasonality

**Fig. 4:**
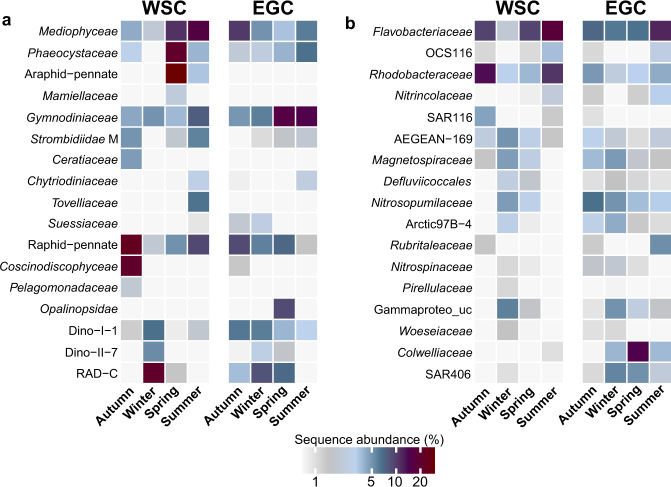
Microbes as indicators for seasons. Relative sequence abundances of major microbial families by season and region (see Supplementary Fig. 5a for details).

**Fig. 5:**
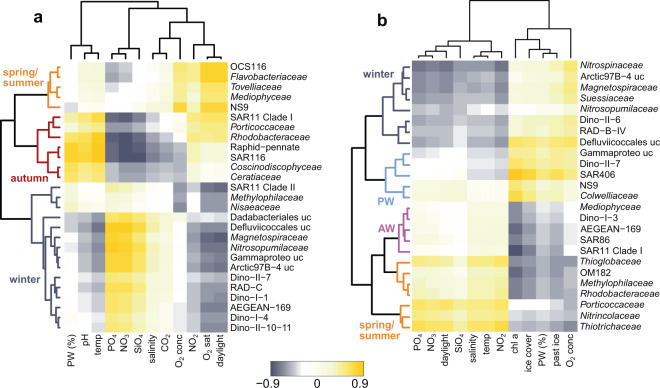
Environmental drivers of community structure. Partial Least Square regression between environmental parameters and the abundance of microbial families, identifying seasonal groupings in the WSC (**a**) compared to both seasonal and polar/Atlantic-influenced groupings in the EGC (**b**). Only correlation coefficients > 0.5 were considered. Temp: water temperature; O_2_ conc: oxygen concentration; O_2_ sat: oxygen saturation; PW: proportion of Polar Water; ice cover: percent ice cover on a given sampling event; past ice: percent ice cover integrated over the time between sampling events; CO2: partial CO_2_ pressure.

We contextualized major patterns in microbial and physicochemical variability (Figs. 2−4) to delineate the four seasons: spring (mid-April to mid-June), summer (mid-June to late-July), autumn (August to October), and winter (November to mid-April). Comparing all sampling events in the WSC and EGC, community structures largely clustered by season, with up to ~60% compositional dissimilarity to the other seasons respectively. Nonetheless, region-specific subclusters underlined the influence of hydrographic differences on microbiome composition (Supplementary Fig. 4). Seasonal contrasts in physicochemistry (Fig. 3a, Supplementary Table 3) and community composition (Fig. 3b, Fig. 4, Supplementary Fig. 5a) were most pronounced in the WSC, corroborated by season-specific correlations between microbial taxa and environmental parameters (Fig. 5). Weaker seasonality in the EGC corresponded to the combined influence of deeper sampling depth, sea ice cover, and the proportions of PW (Figs. 3b, 5). In line with recent metagenomic evidence, these patterns indicate a considerable degree of temporal specialization among Fram Strait microbiomes [51, 52], although the abundant SAR11 and SAR86 clades (constituting on average 25 ± 6% and 8 ± 3% of sequences, respectively) varied little over the year (Supplementary Fig. 5a).

In the WSC, daylight and temperature were significant drivers of eukaryotic variability (PERMANOVA, *p* < 0.001), whereas bacterial composition varied mostly with temperature (PERMANOVA, *p* < 0.001), comparable to the global TARA microbiome study [53]. Bacterial alpha-diversity peaked at the end of polar night (Fig. 2b) when water temperatures were lowest (Fig. 1b), underlining the day-night shift as key transition event. ASVs associated with Bacillariophyta (i.e. diatoms) and Flavobacteriales predominated from spring to autumn (Fig. 2a), presumably corresponding to metabolic interrelations through algal carbohydrates [54]. In contrast, heterotrophic eukaryotes (foremost Syndiniales and RAD-C radiolarians), archaea (Nitrosopumilales) and specific bacterial taxa (e.g., Rhodospirillales) prevailed in winter, with additional short-lived peaks of the diatom parasites *Pirsonia* and MAST-3 (Fig. 2a). We consider these taxa as “microbial recyclers” persisting on detrital, inorganic or semi-refractory substrates. For instance, as detailed below, Nitrosopumilales are involved in ammonia oxidation and hence nitrate replenishment. The separation into photoautotrophy- and heterotrophy-driven periods of production and recycling was reflected in nutrient concentrations, with depletion in summer and replenishment during winter (Fig. 1b, Supplementary Table 1).

In the EGC, changes between polar and Atlantic conditions caused more variable community composition, turnover and diversity. For instance, environmental conditions during AW advection in January resembled those in August (Figs. 1b, 3a). Daylight, temperature, hydrography and ice cover all contributed to microbial community structuring (PERMANOVA, *p* < 0.05). This explained why some taxa correlate with seasonally changing environmental parameters, and some with polar or Atlantic conditions (Fig. 5). Constant proportions of photoautotrophic and heterotrophic eukaryotes year-round, with ~50% lower diatom abundances than in the WSC (Fig. 2a, Supplementary Fig. 5b), illustrated a more heterotrophic food web largely determined by sampling depth [55]. Sensor data available from autumn 2017 onwards show that <1% of photosynthetically active radiation reaches 80 m, impeding primary production. Furthermore, stratification in the upper ~50 m is strong [33, 34]. Detected phytoplankton sequences thus largely correspond to sinking cells from surface blooms and ice [56, 57]. In this context, high ice cover between May and July presumably repressed light availability and hence surface primary production, while stimulating the downward flux of ice-derived microbes. This combination of factors contributed to the weaker seasonality and temporal lag in the detection of certain phytoplankton taxa. For instance, *Phaeocystaceae* and *Mediophyceae* primarily occurred in summer/autumn (EGC) compared to spring/summer (WSC) respectively (Fig. 4). Nonetheless, our results indicate some overarching seasonal principles, especially during AW recirculation to the EGC. In the following, we present a detailed synopsis of seasonal patterns and specific events in chronological order from autumn 2016 to summer 2017.

### Autumn

**Fig. 6:**
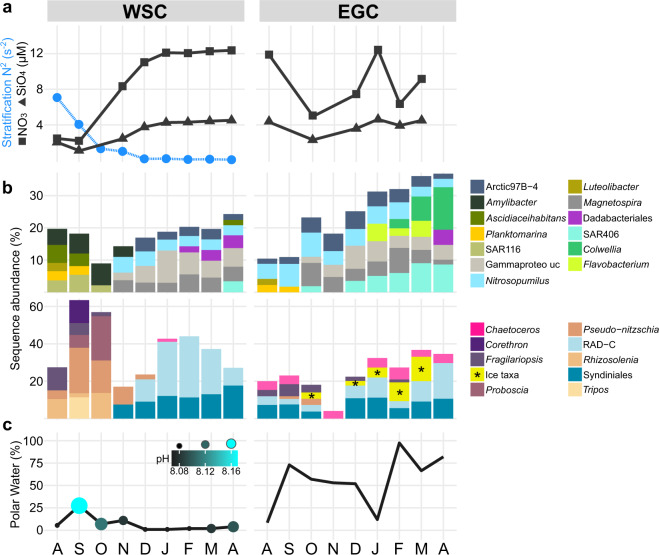
Autumn and winter dynamics. **a** Concentrations of nitrate (squares) and silicate (triangles) in relation to stratification (blue; only available for the WSC). **b** Microbial genera with increased proportions in autumn or winter. “Winter-ice” eukaryotes are combined (marked by asterisks; see Supplementary Fig. 7a for abundances of each genus). **c** pH values (only available for the WSC) and proportions of Polar Water.

Autumn in the WSC was characterized by nitrate, silicate and phosphate depletion and a specific community of *Coscinodiscophyceae*, *Ceratiaceae*, SAR116 and *Rhodobacteraceae* (Figs. 3, 4−6, Supplementary Table 3). These patterns illustrate a post-bloom state, with growing decay of summer phytoplankton [58] and concurrent increase in mixotrophic dinoflagellates [59]. The prevalence of *Corethron*, *Rhizosolenia* and *Proboscia* sequences (Fig. 6b, Supplementary Fig. 5b) matched microscopic cell counts [60], corroborating our amplicon-based results. Similar autumn patterns in the Southern Ocean indicate bi-polar seasonal preferences of *Coscinodiscophyceae*, likely facilitated by their ability to overcome silicate limitation [61], use ammonium instead of nitrate [17], and resist grazing [62]. Appearance of chytrid fungi and *Labyrinthulaceae* at maximal nutrient depletion in October (Supplementary Fig. 6) indicates saprophytic activity on decaying algae [63, 64]. Up to 13-fold higher abundances of *Cand*. Puniceispirillum, other SAR116 members as well as *Ascidiaceihabitans, Amylibacter and Planktomarina* (Fig. 6b) were probably fueled by DMSP and senescence compounds from decaying phytoplankton [65, 66]. Detection of *Luteolibacter* from the *Rubritaleaceae* family (Fig. 5b) mirrored autumn in coastal Svalbard [67] and suggested ongoing particle formation, typical processes in ageing phytoplankton [68]. Overall, the average mixed layer depth of 17 m (Supplementary Table 3) suggests that microbial signals partially correspond to cells sinking from the shallow productive layer.

*Fragilariopsis* co-occurred in the WSC and the EGC during early autumn (Fig. 5b). We hypothesize that this typically ice-associated taxon was transported to the WSC by advection, considering the higher proportion of PW during this time (Fig. 6c). This event also covaried with higher pH, with potential metabolic effects on prevalent taxa such as *Pseudo-nitzschia* [69]. Otherwise, the EGC displayed quite different dynamics. Peaking diatom abundances characterized autumn as major photosynthetic period (Fig. 4, Supplementary Fig. 5b). We attribute this delay to the low ice cover (Fig. 1b, Supplementary Table 3) enhancing light penetration and stratification [70]. This combination presumably allowed an autumn surface bloom, becoming subsequently detectable at 80 m once phytoplankton cells sank.

### Winter

The WSC and EGC shared elevated abundances of *Magnetospiraceae*, *Nitrospinaceae*, the Arctic97B-4 clade and unclassified Gammaproteobacteria (Figs. 4, 5b), although their winter-summer contrasts were stronger in the WSC (average Kruskal-Wallis significance *p* ≤ 0.003 vs. 0.02 in the EGC). Furthermore, Dadabacteriales appeared from February (WSC) or late March (EGC) (Fig. 6) and might contribute to the recycling of organic matter [71]. Fundamental regional differences were the complete switch to heterotrophy in the WSC, compared to ice-related microbial signatures including persistent diatom signals in the EGC.

#### Heterotrophic winter communities of the WSC

The increase of Syndiniales, parasitic recyclers of phytoplankton biomass [72], in November marked the onset of winter (Supplementary Fig. 6). Bacterial diversification and nutrient replenishment (Figs. 2, 6) followed the breakdown of summer stratification, with maximal mixing of the water column in January (Fig. 6a). At this time, heterotrophic eukaryotes constituted ~70% of sequences and nutrient standing stocks were restored (Figs. 2a, 6a). The parallel decline of phototrophs to a combined relative abundance of <5% (Supplementary Fig. 5b) indicated complete mixing as one central turning point of the annual cycle [73, 74]. Notably, this also illustrates that only a small “seed bank” overwintered to initiate the following spring bloom. The upward transport of microbes during mixing likely enriched the community’s metabolic potential [75]. For instance, appearance of deep-water RAD radiolarians [76] possibly contributed to the recycling of phytoplankton biomass. Stratification potentially also influenced the temporal succession of different Syndiniales lineages over winter (Supplementary Fig. 6).

Winter bacteria and archaea likely contributed to nutrient replenishment. The co-occurrence of *Nitrosopumilaceae* and *Nitrospinaceae* (Figs. 4, 6b), the major drivers of marine nitrification, suggests an interactive niche with initial oxidation of ammonia or urea by *Nitrosopumilaceae* and subsequent nitrite oxidation by *Nitrospinaceae* [77]. In addition, the *Magnetospiraceae* family (Rhodospirillales) might recycle nitrogen by fixation and contribute to a yet underestimated nitrogen source [78, 79]. Furthermore, metaproteomic data indicate that *Magnetospiraceae* perform CO_2_ fixation and thiosulfate oxidation [13]. Overall, genomic and metabolic evidence suggests consistent roles of *Nitrosopumilaceae*, *Nitrospinaceae*, and *Magnetospiraceae* during winter in both Arctic and Antarctic Oceans [80, 81]. Further potential recyclers are the *Pirellulaceae* and *Woeseiaceae* through ammonia oxidation and denitrification respectively [82, 83]. The winter niche of Defluviicoccales was potentially fueled by stored glycogen or unsaturated aliphatics [84, 85]. Overall, the prevalence of diverse heterotrophic and chemoautotrophic taxa illustrates the polar night as important recycling phase before the spring bloom. Furthermore, the winter microbiome is not static, but responsive to certain stimuli such as mixing.

#### An ice-related microbial loop in the EGC

Unique to the EGC was the persistence of raphid-pennate diatoms and flavobacteria throughout winter (Fig. 4), contrasting their light-correlated seasonality in the WSC. We attribute these signals to ice melt and release of cells into the water, following intermittent water temperatures of >2 °C during AW advection in January (Fig. 1b). The diatoms *Bacillaria* and *Naviculales*, together with *Polarella* and *Chrysophyceae* flagellates, constituted up to 15% of sequences between February and March (Fig. 6b, Supplementary Fig. 7a). All of these taxa occur in sea ice and the underlying water [56, 86], possibly constituting an ice-related microbial loop. Ice algae produce copious amounts of storage polysaccharides and extracellular polymeric substances, fueling bacterial growth in the underlying water [70, 87]. *Bacillaria* exudates are a valuable nutrient source for bacteria [88], as is chrysolaminarin from diatoms and *Chrysophyceae* [89]. Concurrently, *Chrysophyceae* potentially also influenced organic matter cycling by preying on bacteria [90]. A *Flavobacterium* ASV constituted ~10% between January and March (Fig. 6b, Supplementary Fig. 7a), sharing >99% sequence similarity with *Flavobacterium frigidarium*, a psychrophilic genus with laminarinolytic abilities [91]. Detection of related sequences on ice-algal aggregates [92] supports a presumed niche of this ASV through utilization of ice-algal carbohydrates. Overall, such ice-fueled processes might explain signatures and activities of specific microbial taxa in the warming Arctic [93, 94].

**Fig. 7:**
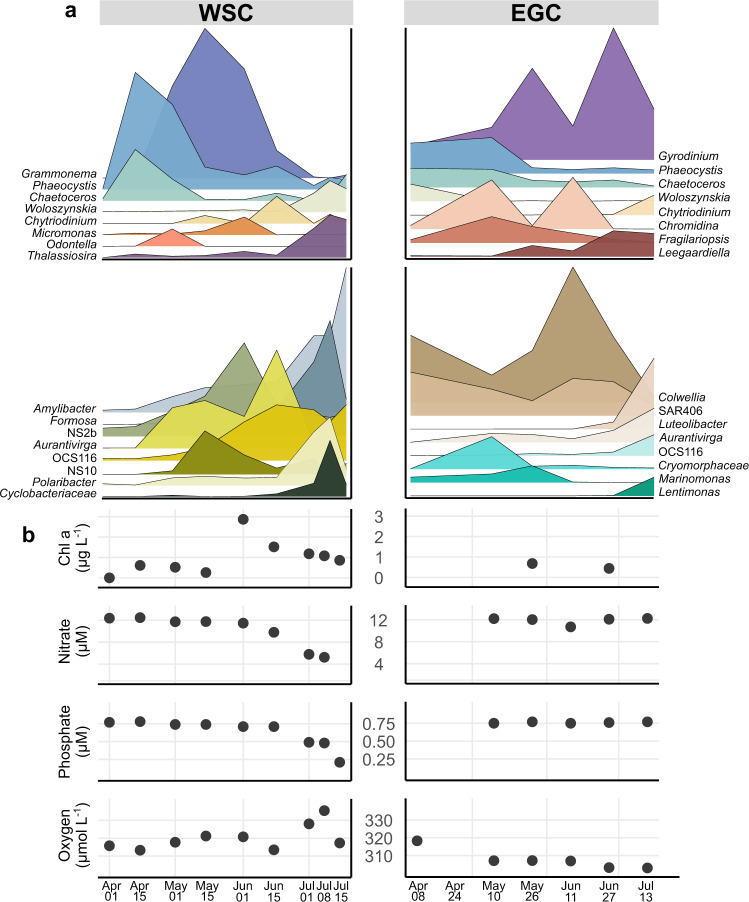
Spring and summer dynamics. **a** Relative abundances of dominant eukaryotic and bacterial genera (see Supplementary Fig. 8 for detailed abundances). **b** Concentrations of chlorophyll, nitrate, phosphate and oxygen.

An EGC-specific winter bacterium was the SAR406 clade, peaking at 9% sequence abundance in March and remaining detectable into summer. In addition, the frequently ice-associated genus *Colwellia* increased from February to abundances of >20% in mid-June (Figs. 6b, 7a). Both SAR406 and *Colwellia* markedly correlated with ice cover (Spearman’s *rho* = 0.7, *p* < 0.0004), suggesting that ice cover sustained these winter-type taxa into summer. As SAR406 might participate in sulfur cycling [95], loss of sea ice might diminish the recycling of inorganic substrates.

### Spring and summer

#### Microbial succession in the WSC

Once daylight reached ~20 h in mid-April, the microbial system returned to a phototrophic state. The winter-spring transition occurred within few weeks, comparable to warmer Pacific waters [96]. The average mixed layer depth of >200 m until mid-June (Supplementary Table 3) likely facilitated strong phytoplankton growth. Eukaryotic composition changed ahead of bacterial communities, whose structure changed within four weeks after the primary photosynthetic peak (Supplementary Fig. 6). We observed three distinct bloom stages, featuring phototrophic pioneers *(Phaeocystis* and *Chaetoceros*) followed by araphid-pennate diatoms (*Grammonema*) and centric diatoms (*Thalassiosira*) (Fig. 7, Supplementary Fig. 8). A comparable three-stage bloom has been observed a year before in nearby Kongsfjorden [97]. The replacement of eukaryotic heterotrophs by photoautotrophs (Fig. 3b, Supplementary Fig. 6) suggests considerable energy fluxes around the winter-spring transition, with possible effects on benthopelagic coupling [98–100]. The early detection of *Aurantivirga* and SAR92 (Supplementary Fig. 6) matched observations during the Antarctic spring bloom [17], indicating comparable temporal niches at both poles. The *Grammonema* abundance of >50% in May coincided with peaking chlorophyll, potentially fueling intermittent peaks of *Formosa*, *Polaribacter*, and NS clades from family *Flavobacteriaceae* (Fig. 7a), comparable to diatom-flavobacteria relationships in temperate and Antarctic waters [54, 101].

*Thalassiosira* was specific for summer and the final bloom stage, when nitrate and phosphate declined and oxygen concentrations peaked (Fig. 7). The average mixed layer depth in summer was 23 m (Supplementary Table 3); hence, the RAS sampled just below the productive layer. The relative increase of mixotrophic flagellates (e.g., *Gyrodinium* and *Woloszynskia*) and concurrently decreasing chlorophyll indicates that trophic structure shifted towards heterotrophy. Increase of the roseobacter *Amylibacter* (formerly NAC11-7) to 15% sequence abundance emphasized the beginning transition to the autumn post-bloom where *Rhodobacteraceae* dominated (Fig. 4). We hypothesize concurrent generation of detritus particles, given the typical termination of diatom blooms by aggregation [68] and the association of *Amylibacter* with related particles [102]. Furthermore, the appearance of ectoparasitoid dinoflagellates such as *Chytriodinium* indicates beginning parasitism on diatoms and larger metazoans [103].

#### Absence of major phototrophic peaks in the EGC

Diatom abundances resembled those during winter (Supplementary Fig. 5b), with threefold lower chlorophyll concentrations than the WSC peak (Fig. 7b). *Fragilariopsis* and *Chaetoceros* together only constituted <10% of eukaryotic sequences, although nutrients were not limiting (Fig. 7b, Supplementary Table 3). Furthermore, *Phaeocystis* only reached 9% and hence a quarter of WSC proportions. These observations corroborate the influence of sampling depth, i.e. that phytoplankton sequences merely mirror preceding surface dynamics and export flux. This constant input of detrital material presumably also explains why Syndiniales prevailed over summer (Fig. 4), together with major peaks of the mixotrophs *Chromidina* (Ciliophora) and *Gyrodinium* (Dinoflagellata) that constituted up to 35% of eukaryote sequences. *Chromidina* is normally considered an animal parasite, suggesting yet undescribed free-living niches in the marginal ice zone. The prevalence of mixotrophy was underlined by the earlier detection of *Woloszynskia*, and twofold lower flavobacterial abundances compared to the WSC (Figs. 2a, 4b). Moreover, the typical phytoplankton associates OCS116, *Lentimonas* and *Luteolibacter* [104, 105] were only detected from mid-summer, following EGC-specific *Cryomorphaceae* and *Marinomonas* peaks (Fig. 7a). The presence of ice cover over summer, likely resulting in continuous input of ice-derived substrates, indicates further differences in trophic structure. Ice substrates presumably fueled the major peak of *Colwellia*, which can efficiently grow on organic matter from sea ice [70].

## Ecological conclusions

This first assessment of microbial seasonality in the Fram Strait by autonomous sampling identified marked seasonal contrasts, distinct transition events, as well as dynamic variability linked to polar vs. Atlantic conditions. The characterization of bloom stages, ephemeral abundance peaks, and polar night characteristics promotes the understanding of the drivers and timescales of microbial seasonality in ice-covered and ice-free Arctic waters. These insights yield a number of fundamental ecological conclusions, with implications for the present and future Arctic Ocean.

1. We identified major dynamics and drivers of microbiome structure in the Arctic Ocean: marked seasonal contrasts related to daylight, temperature and stratification in the euphotic zone of the ice-free WSC, compared to weaker seasonality related to ice cover, proportions of polar/Atlantic water masses and sampling depth in the EGC.
2. Dynamics in the WSC illustrate key principles of microbial seasonality in the ice-free, open Arctic Ocean: *Phaeocystis* as daylight pioneer followed by pennate diatoms and maximum chlorophyll concentrations when mixed layer depth was still >200 m (spring); declining nitrate and shift towards centric diatoms and mixotrophic flagellates upon increasing stratification (summer); minimum nutrients and highest temperatures when *Coscinodiscophyceae* diatoms and oligotrophic bacteria prevailed (autumn); and chemoautotrophic microbial recyclers and nutrient replenishment during vertical mixing (winter). Comparable observations have been made in a year-round study using Niskin-based sampling [11], illustrating that autonomous techniques provide results consistent with traditional approaches while considerably increasing temporal resolution. Moreover, our results remarkably overlap with a RAS-based study in the open Southern Ocean, which also reports *Coscinodiscophyceae* in autumn, *Aurantivirga* and SAR92 as first bacterial responders, and *Amylibacter* at the summer-autumn transition [17]. This suggests fundamental “bi-polar” patterns of microbial seasonality, only discernable by autonomous sampling.
3. The EGC exhibited combined effects of depth, ice cover and variable polar/Atlantic water masses, with a strong heterotrophic signature year-round. Seasonality and similarities to the WSC scaled with the extent of AW advection. At a maximum speed of 0.25 m s^−1^ [106], water from the WSC can reach the EGC within ~2 weeks, underlining how quickly hydrographic regimes can change and influence community composition. Polar-dominated conditions extended the duration and abundance of winter taxa such as SAR406 and *Colwellia*, with surface phytoplankton growth mainly detected during low ice in autumn. Periods of low ice coincided with higher abundances of *Phaeocystis*, *Thalassiosira*, OCS116 and *Aurantivirga* (Supplementary Fig. 7b). These dynamics are sentinels of how the future EGC might shift from an ice- to a light-driven habitat [107], presumably affecting the fate of phytoplankton blooms and the biological carbon pump [57, 108, 109]. Elevated photosynthesis and resulting higher amounts of organic substrates might accelerate the microbial loop [110], inducing the remineralization of ice-derived organic matter at the expense of chemoautotrophic metabolisms [70, 111].
4. Atlantification of the Arctic may enhance early blooms of *Phaeocystis* [15, 27] and alter biogeochemical fluxes, considering the associated production of TEP that serves as microbial substrate, microhabitat and downward vehicle of organic matter. In case stratification becomes stronger and more permanent with increasing temperatures, wintertime convection might diminish and deep-water “recycling taxa” disappear from the winter assemblage, with yet unknown ecological consequences.

In conclusion, the demonstrated seasonal microbiome dynamics and drivers contribute to the understanding of Arctic ecosystem functioning over polar day and night. This evidence is particularly relevant considering the anticipated impact of climate change on polar regions.

## Supplementary information

Supplementary Fig. 1

Supplementary Fig. 2

Supplementary Fig. 3

Supplementary Fig. 4

Supplementary Fig. 5

Supplementary Fig. 6

Supplementary Fig. 7

Supplementary Fig. 8

Supplementary Table 1

Supplementary Table 2

Supplementary Table 3

Supplementary Methods
