# Supplementary figures and images for: The polar night shift: seasonal dynamics and drivers of Arctic Ocean microbiomes revealed by autonomous sampling
Source: ISME Commun. 2021 Dec 11;1:76. doi: 10.1038/s43705-021-00074-4 (PMC9723606; doi:10.1038/s43705-021-00074-4)

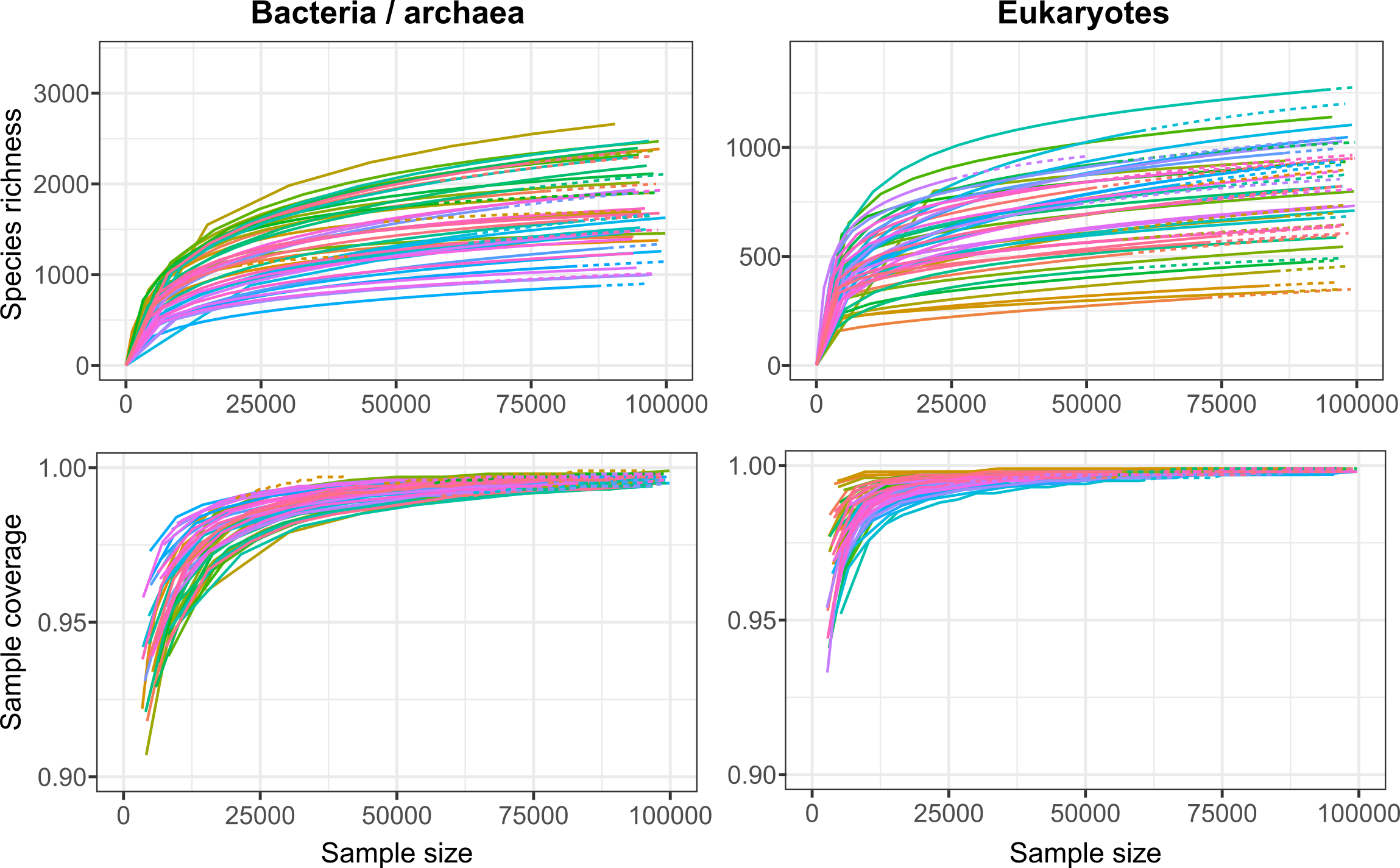

Supplement: Supplementary file 1 — Supplementary Fig. 1 [file 43705_2021_74_MOESM1_ESM.png]

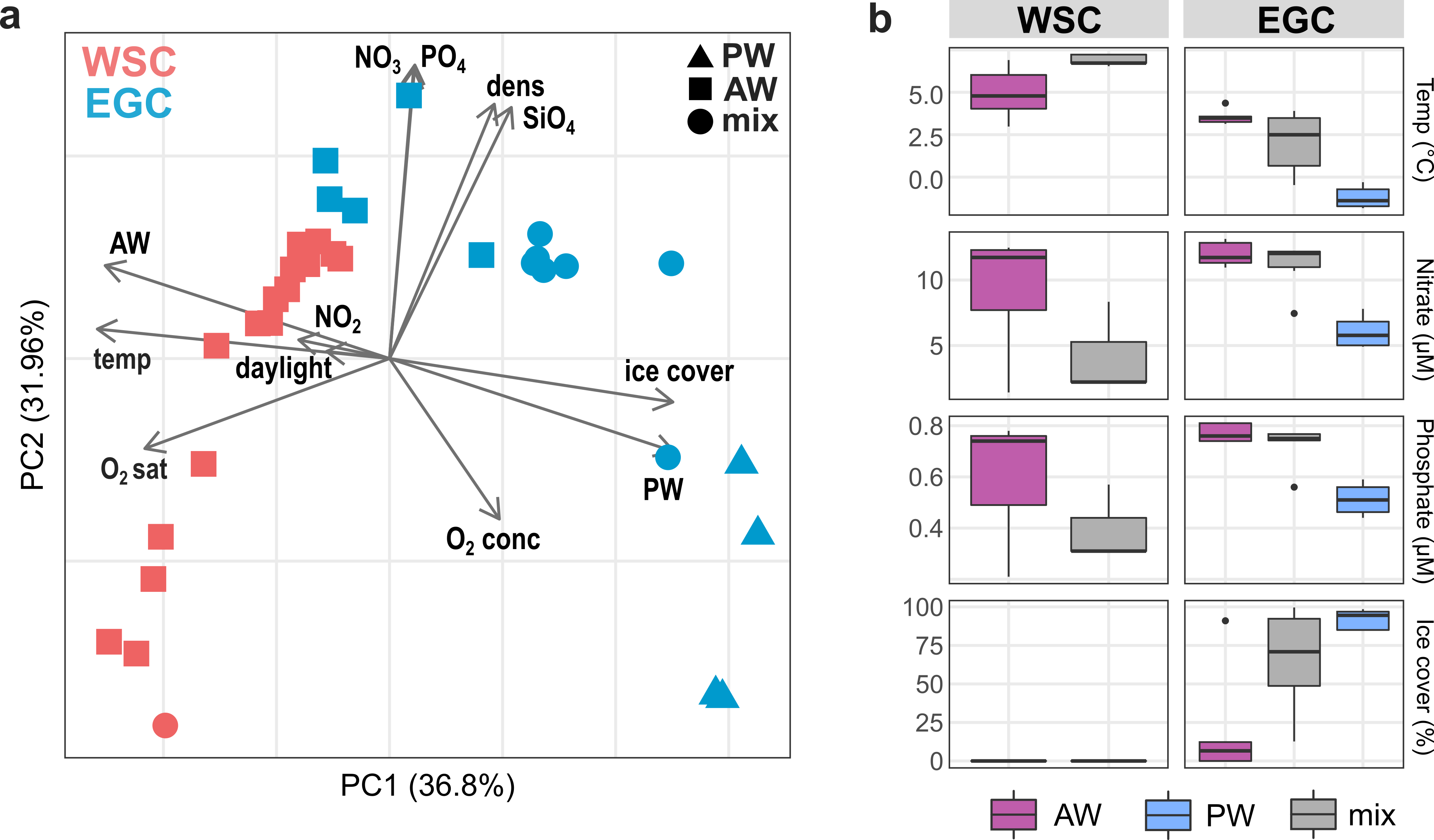

Supplement: Supplementary file 2 — Supplementary Fig. 2 [file 43705_2021_74_MOESM2_ESM.png]

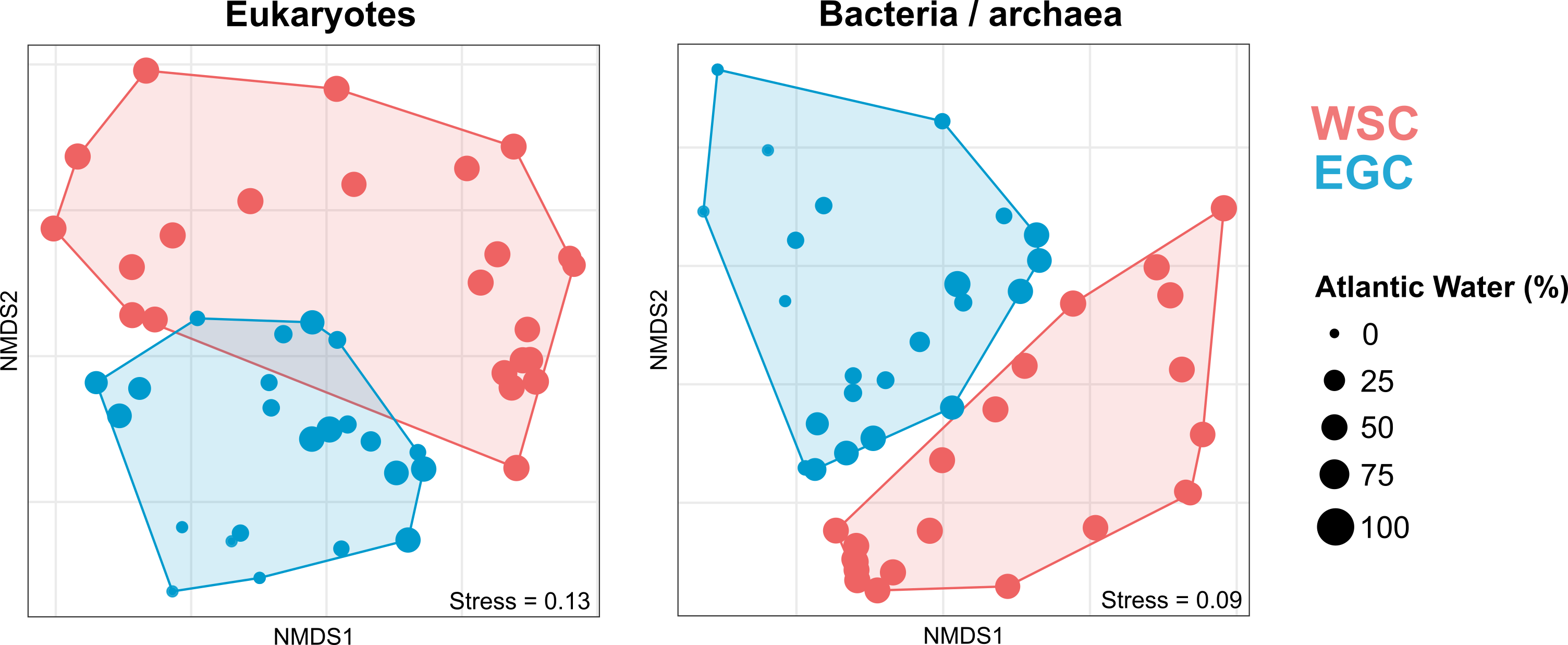

Supplement: Supplementary file 3 — Supplementary Fig. 3 [file 43705_2021_74_MOESM3_ESM.png]

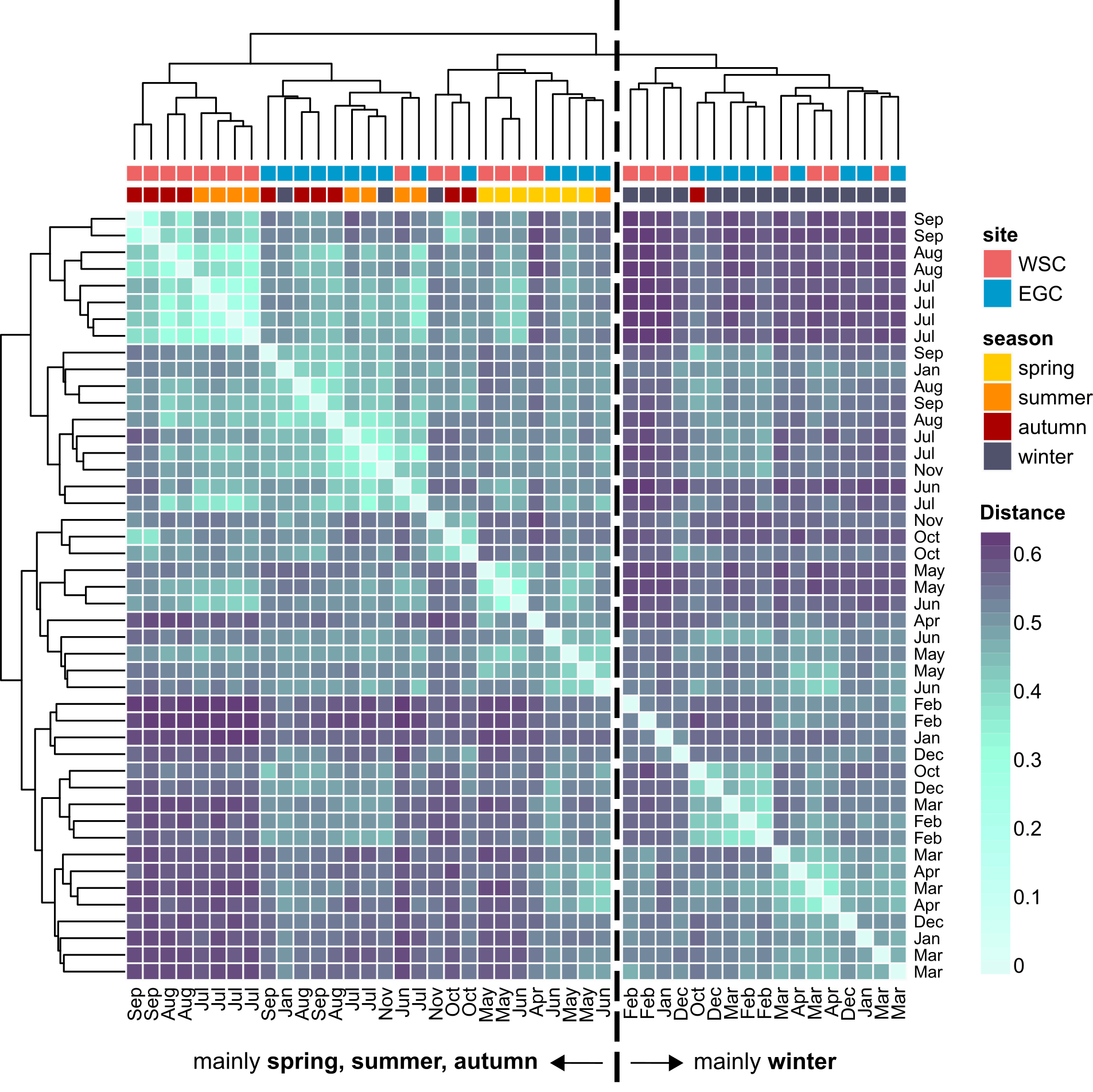

Supplement: Supplementary file 4 — Supplementary Fig. 4 [file 43705_2021_74_MOESM4_ESM.png]

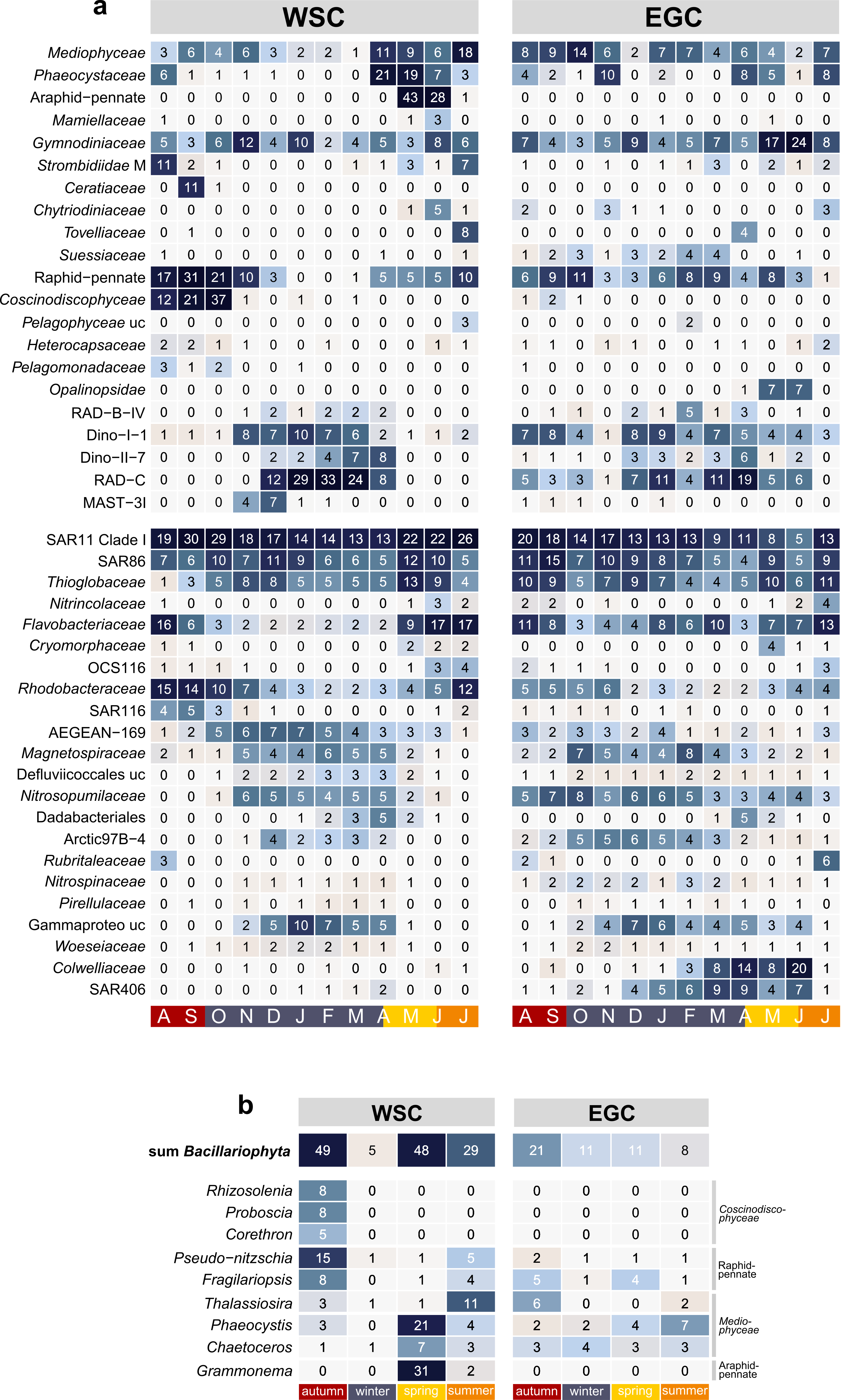

Supplement: Supplementary file 5 — Supplementary Fig. 5 [file 43705_2021_74_MOESM5_ESM.png]

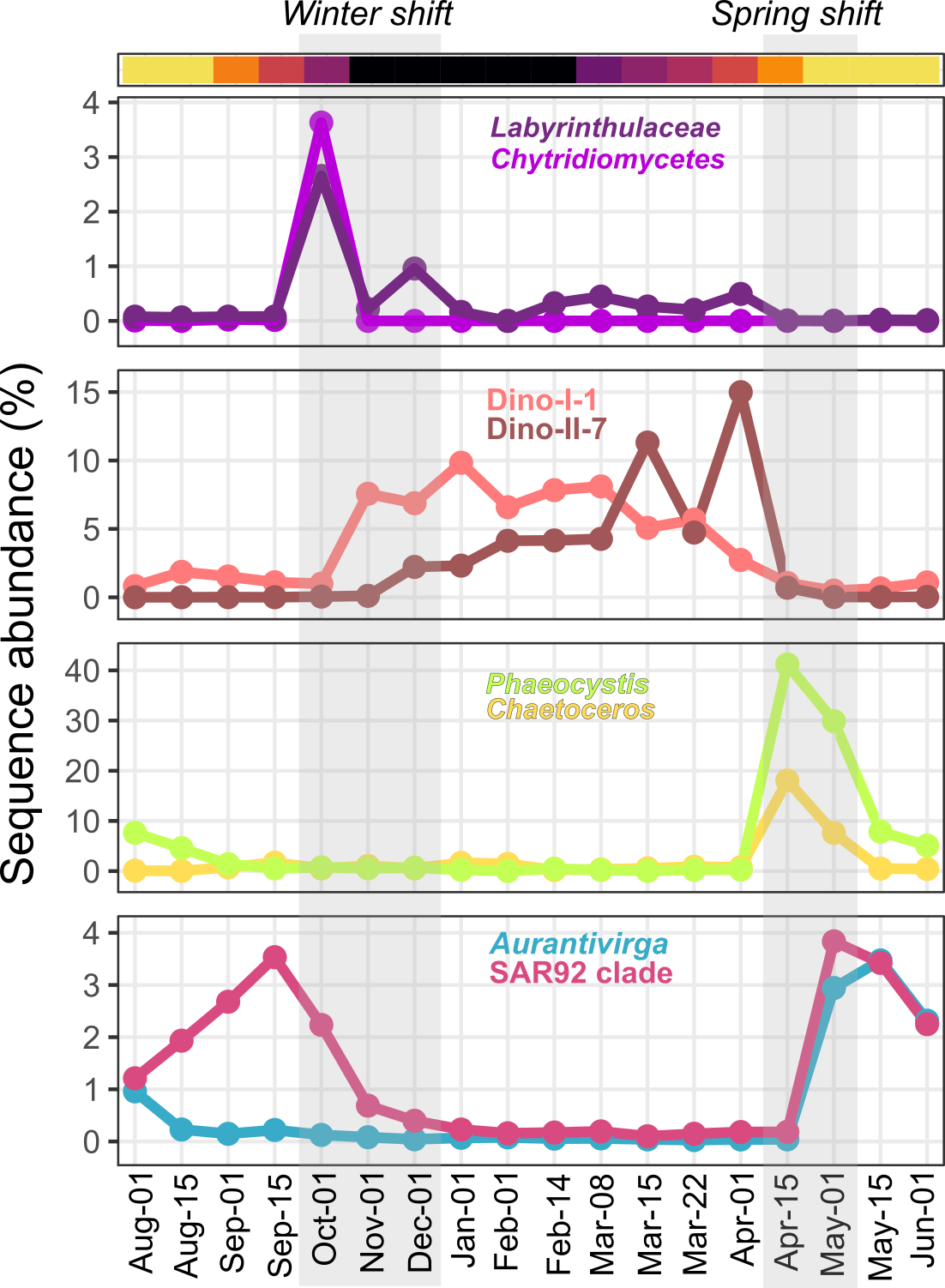

Supplement: Supplementary file 6 — Supplementary Fig. 6 [file 43705_2021_74_MOESM6_ESM.png]

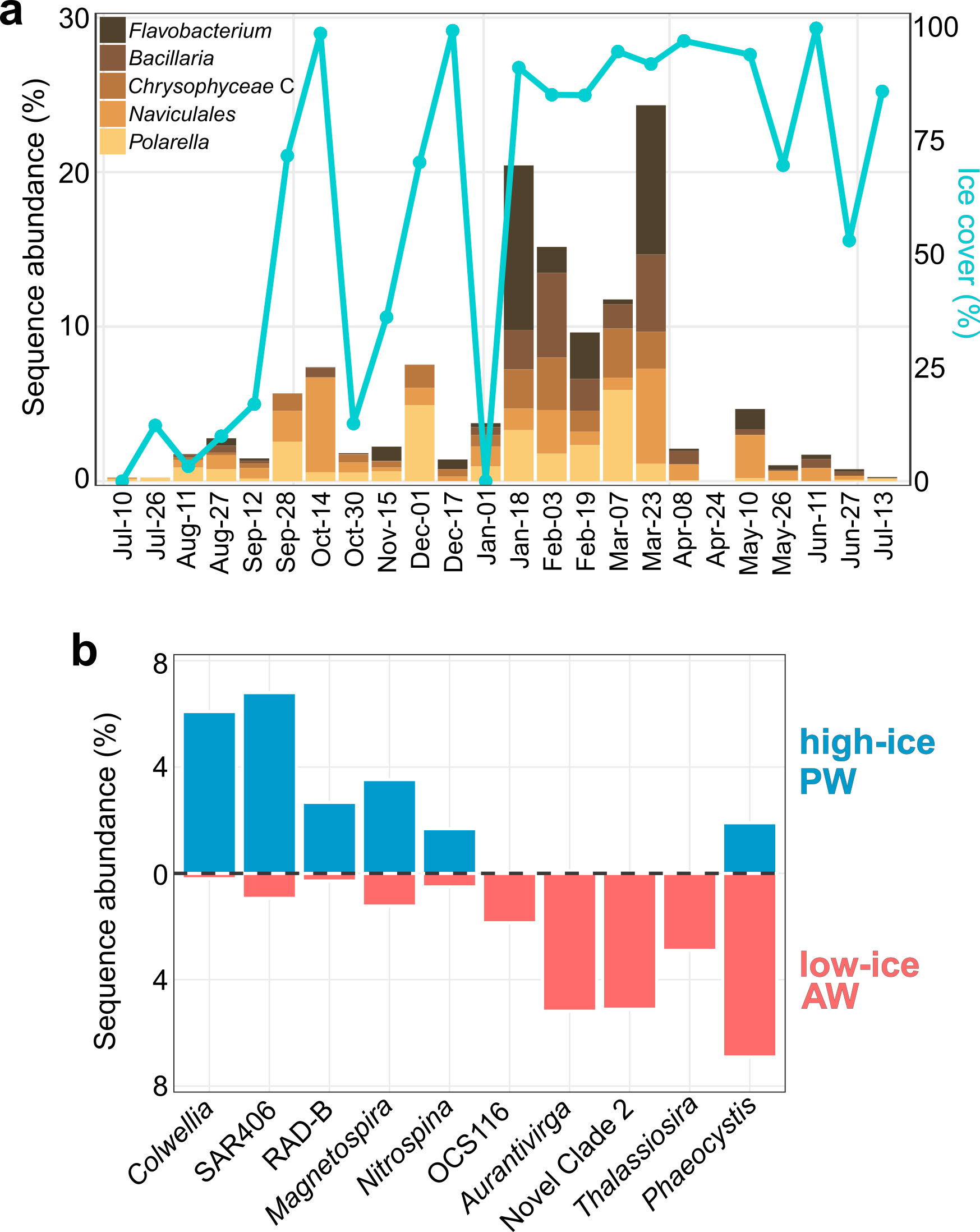

Supplement: Supplementary file 7 — Supplementary Fig. 7 [file 43705_2021_74_MOESM7_ESM.png]

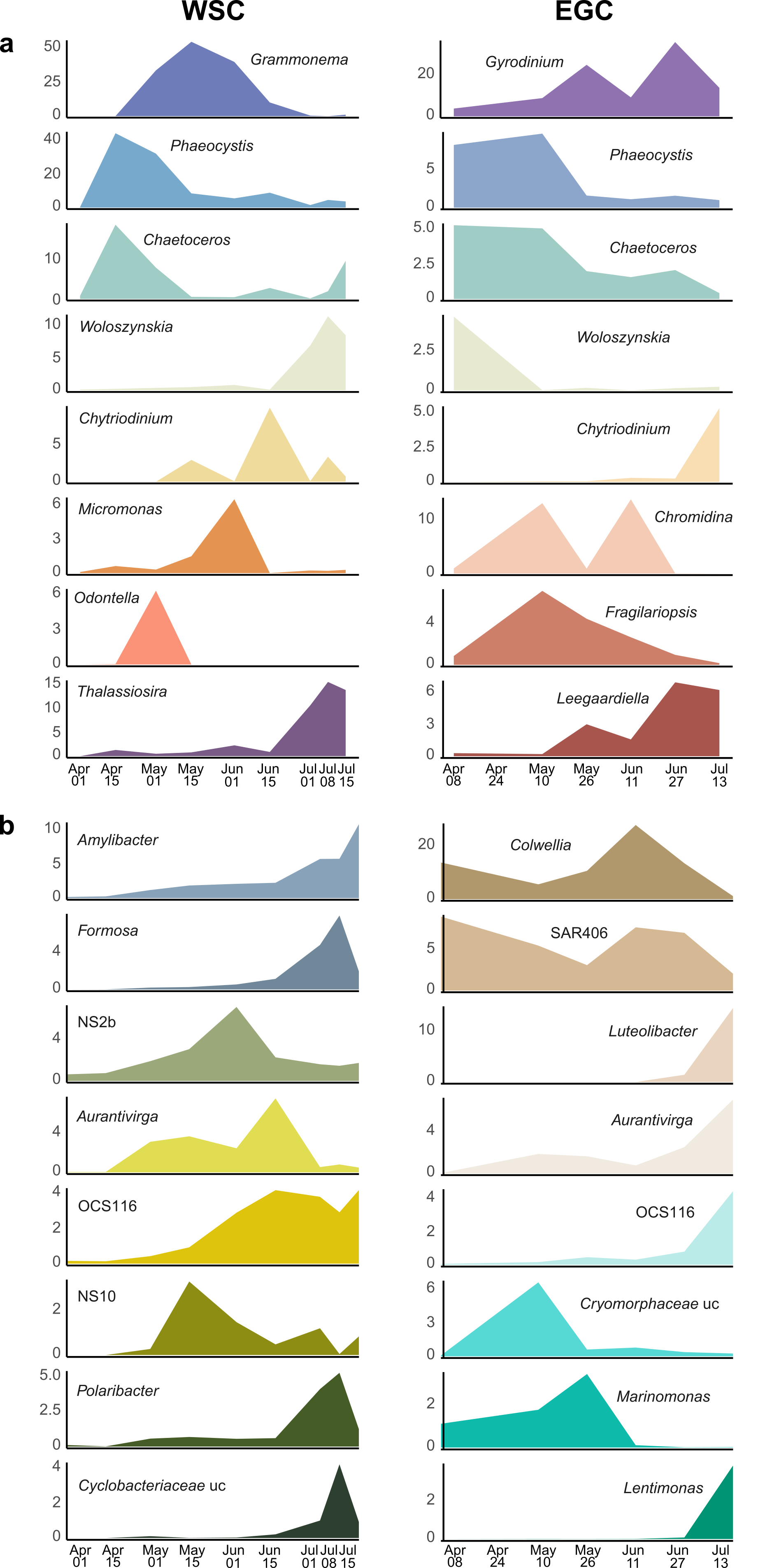

Supplement: Supplementary file 8 — Supplementary Fig. 8 [file 43705_2021_74_MOESM8_ESM.png]
