# Supplementary material for: The polar night shift: seasonal dynamics and drivers of Arctic Ocean microbiomes revealed by autonomous sampling
Source: ISME Commun. 2021 Dec 11;1:76. doi: 10.1038/s43705-021-00074-4 (PMC9723606; doi:10.1038/s43705-021-00074-4)
Supplement: Supplementary file 12 — Supplementary Methods [file 43705_2021_74_MOESM12_ESM.pdf]

The polar night shift: Seasonal dynamics and drivers of Arctic Ocean microbiomes revealed by autonomous sampling

Matthias Wietz, Christina Bienhold, Katja Metfies, Sinhué Torres-Valdés, Wilken-Jon von Appen, Ian Salter, Antje Boetius

SUPPLEMENTARY METHODS

Illumina amplicon sequencing

Library preparation was performed according to the standard instructions of the 16S Metagenomic Sequencing Library Preparation protocol (Illumina, San Diego, CA). The hypervariable V4–V5 region of bacterial 16S rRNA genes was amplified using primers 515F (GTGYCAGCMGCCGCGGTAA) and 926R (CCGYCAATTYMTTTRAGTTT). The hypervariable V4–V5 region of eukaryotic 18S genes was amplified using primers 528iF (GCGGTAATTCCAGCTCCAA) and 926iR (ACTTTCGTTCTTGATYRR). Sequences were obtained on an Illumina MiSeq platform in 2x300 bp paired-end runs at CeBiTec (Bielefeld, Germany) or Alfred Wegener Institute following the standard instructions of the 16S Metagenomic Sequencing Library Preparation protocol (Illumina). Primer-clipped reads were processed into amplicon sequence variants (ASVs) following the standard DADA2 workflow at https://benjjneb.github.io/dada2/tutorial.html [1]. Filtering settings for 16S rRNA amplicons were truncLen=c(230,195), maxN=0, minQ=2, maxEE=c(3,3) and truncQ=0, followed by merging using minOverlap=10 and chimera removal. Filtering settings for 18S rRNA amplicons were truncLen=c(250,200), maxN=0, minQ=2, maxEE=c(3,3) and truncQ=0, followed by merging using minOverlap=20 and chimera removal. After singleton removal, we obtained on average 62,000 16S rRNA and 99,000 18S rRNA reads per sample (Supplementary Table 2) that sufficiently covered community composition (Supplementary Fig. 1). 16S and 18S ASVs were taxonomically classified using the Silva v138 [2] and PR^2 v4.12 [3] databases respectively. Data was processed and visualized using R packages tidyverse, phyloseq, ampvis2 and PNWColors [4–7], with aesthetic modifications of figures using Inkscape (https://inkscape.org). The complete amplicon workflow is available under https://github.com/matthiaswietz/RAS-1617.

Characterization of water masses

Water masses were characterized following Richter and colleagues [8]. Unlike in that study, only warm Atlantic Water but not Arctic Atlantic Water play a role in our case. As the three types of Atlantic Water (wAW, AW, DW) are temperature-stratified, it is not expected that wAW and DW directly mix without AW in-between. Therefore, two different end member triangles

involving only one of wAW and DW can be solved. We define four water masses: warm Atlantic Water (wAW, salinity 35.16, 8°C), Atlantic Water (AW, salinity 35.1, 4.1°C), Polar Surface Water (PSW, salinity 34.17, −1.8°C) and Deep Water (DW, salinity 34.93, −0.9°C). We first applied an end-member decomposition to a wAW-AW-PSW triangle. For some measurements, the solution falls into that triangle meaning that all fractions are between 0 and 1. A number of data points fall to the top/left of the triangle as indicated by negative AW fractions, which we kept as such. A further amount of data points fall to the bottom/right of the triangle, i.e. in the majority into the DW-AW-PSW triangle as indicated by negative wAW fractions. For these cases, we set the wAW fraction to 0 and solve the DW-AW-PSW fractions. This provides four time series for the water masses where at each time point either the wAW or the DW fraction is 0. The total AW fraction (tAW) is defined as the sum wAW+AW+DW. This combines with the PSW time series to add to 1. At times when the water is fresher than PSW, the PSW fraction is > 1 and the tAW is < 0. In those cases, we set the respective values to 1 and 0. Likewise for the cases where tAW > 1. While keeping the details of this calculation in mind, the Polar Water fraction closely follows times of low salinity.

SUPPLEMENTARY MATERIAL

Supplementary Fig. 1 – Rarefaction and coverage analyses of amplicon sequence variants, showing that bacterial, archaeal and eukaryotic community composition were sufficiently covered. Each colored line corresponds to an individual sample.

Supplementary Fig. 2 – Regional variability of environmental conditions. a: Principal Component Analysis of environmental parameters in the WSC (red) and EGC (blue). Symbol shape designates the prevailing water mass at each sampling event. Arrows illustrate the influence of parameters on multivariate clustering. Temp: water temperature; O_2 conc: oxygen concentration; O_2 sat: oxygen saturation; AW: proportion of Atlantic Water, PW: proportion of Polar Water; ice: percent ice cover. **b:** Physicochemical parameters with significant differences between AW, PW and mixed water masses (Kruskal-Wallis test, $p < 0.03$).

Supplementary Fig. 3 – Regional variability of microbial communities. Non-metric multidimensional scaling of Hellinger-transformed relative abundances in the WSC (red) and EGC (blue). Dot size corresponds to the proportion of Atlantic Water.

Supplementary Fig. 4 – Taxonomic similarities of bacterial and archaeal communities between sampling events and sites expressed as Jenson-Shannon distances.

Supplementary Fig. 5 – Detailed overview of microbial community structure. a: Sequence abundances of major bacterial, archaeal and eukaryotic families by month in the

WSC and EGC**. b:** Sequence abundances of major diatom genera by season in the WSC and EGC.

Supplementary Fig. 6 – Seasonal transitions in the WSC in relation to daylight hours (top color gradient).

Supplementary Fig. 7 – EGC-specific patterns. a: Microbial dynamics following the ice minimum after intermittent AW advection in January. **b**: Relative sequence abundances of selected genera in polar (high-ice, cold, lower-nutrient) and Atlantic (low-ice, warmer, higher-nutrient) conditions.

Supplementary Fig. 8 – Detailed abundances of eukaryotic (a) and bacterial (b) genera during spring and summer.

Supplementary Table 1 – Sampling events, measured environmental parameters, and accession numbers of raw sequence files.

Supplementary Table 2 – Amplicon read counts at each major step of the DADA2 pipeline.

Supplementary Table 3 – Seasonal physicochemical patterns in the WSC and EGC.

SUPPLEMENTARY REFERENCES

1. Callahan BJ, McMurdie PJ, Rosen MJ, Han AW, Johnson AJA, Holmes SP. DADA2: High-resolution sample inference from Illumina amplicon data. *Nat Methods* 2016; **13**: 581–583.
2. Quast C, Pruesse E, Yilmaz P, Gerken J, Schweer T, Yarza P, et al. The SILVA ribosomal RNA gene database project: improved data processing and web-based tools. *Nucleic Acids Res* 2013; **41**: D590-596.
3. Guillou L, Bachar D, Audic S, Bass D, Berney C, Bittner L, et al. The Protist Ribosomal Reference database (PR^2): a catalog of unicellular eukaryote Small Sub-Unit rRNA sequences with curated taxonomy. *Nucleic Acids Res* 2013; **41**: D597–604.
4. Wickham H, Averick M, Bryan J, Chang W, McGowan LD, François R, et al. Welcome to the Tidyverse. *J Open Source Softw* 2019; **4**: 1686.
5. McMurdie PJ, Holmes S. phyloseq: an R package for reproducible interactive analysis and graphics of microbiome census data. *PLOS ONE* 2013; **8**: e61217.
6. Andersen KSS, Kirkegaard RH, Karst SM, Albertsen M. ampvis2: an R package to analyse and visualise 16S rRNA amplicon data. *bioRxiv* 2018; 299537–299537.
7. Lawlor J. 2020. PNWColors: color palettes inspired by nature in the US Pacific Northwest. https://CRAN.R-project.org/package=PNWColors
8. Richter ME, von Appen W-J, Wekerle C. Does the East Greenland Current exist in the northern Fram Strait? *Ocean Science* 2018; **14**: 1147–1165.
